# Landfill Leachate Toxicity Removal in Combined Treatment with Municipal Wastewater

**DOI:** 10.1100/2012/202897

**Published:** 2012-04-24

**Authors:** J. Kalka

**Affiliations:** Environmental Biotechnology Department, Silesian University of Technology, 44100 Gliwice, Poland

## Abstract

Combined treatment of landfill leachate and municipal wastewater was performed in order to investigate the changes of leachate toxicity during biological treatment. Three laboratory A2O lab-scale reactors were operating under the same parameters (Q-8.5–10 L/d; HRT-1.4–1.6 d; MLSS 1.6–2.5 g/L) except for the influent characteristic and load. The influent of reactor I consisted of municipal wastewater amended with leachate from postclosure landfill; influent of reactor II consisted of leachate collected from transient landfill and municipal wastewater; reactor III served as a control and its influent consisted of municipal wastewater only. Toxicity of raw and treated wastewater was determinted by four acute toxicity tests with *Daphnia magna, Thamnocephalus platyurus, Vibrio fischeri,* and *Raphidocelis subcapitata*. Landfill leachate increased initial toxicity of wastewater. During biological treatment, significant decline of acute toxicity was observed, but still mixture of leachate and wastewater was harmful to all tested organisms.

## 1. Introduction

Waste volume is growing faster than the world's population, and management of wastes is a matter of considerable human concern [1, 2]. Recycling and recovery of materials and energy are encouraged so as to safeguard natural resources and obviate wasteful use of land [3]. Nevertheless landfilling is still widely accepted and popular method for the ultimate disposal of solid waste material. It is estimated that 90% of solid waste in Poland is disposed of in landfill sites [2]. The internal biochemical decomposition processes taking place within a landfill play a crucial role in determining potential adverse impacts that landfills may have during and beyond its active life. Rainfall and other precipitation percolating through layers of waste may dissolve and wash out products of biochemical processes creating landfill leachate. Many studies have shown that landfill leachate consisted of different groups of pollutants such as organics: alkenes, aromatic hydrocarbons, acids, esters, alcohols, hydroxybenzene, amides, and so forth, as well as ammonia nitrogen and heavy metals. Some authors report that more than 190 substances were identified in leachate, making barely 1% of materials calculated from total organic carbon concentration [4]. Chemical composition of leachate changes with the time span of landfill operation. Typical leachate COD for the transient landfills (2–5 years of operation) is 500–10000 mg/L, while the same parameter for old landfill leachate is less than 500 mg/L [5]. Leachate may endanger aquatic environment due to uncontrolled overflow, subsidence, and infiltration [6–9]. Due to its high organic matter content, landfill leachate was the subject of many research experiment involving advanced oxidation processes (e.g., Fenton, electro-Fenton) as a treatment method [10, 11]. However, the most common practice to avoid risk of contamination is to discharge leachate into wastewater stream and subsequent treatment in wastewater treatment plant. Refractory micro- and macropollutants may pass biological treatment plant unchanged and contribute to still high toxicity of the effluent. It is well known that toxicity of environmental samples (like wastewater or leachate) is a consequence of numerous contaminants, their synergistic or antagonistic effects, and physicochemical properties. As the composition of leachate is unstable during the landfill operation period, adverse effect of leachate is also variable in different operational period. The aim of the present study was to investigate the change of toxicity of landfill leachate in function of the landfill age. Leachate was sampled from landfills of different age (I: postclosure landfill; II: new landfill 2 years of operation), and its toxicity was tested towards selected aquatic organisms. Leachate (separately) was subsequently mixed in different ratio with municipal wastewater and treated in lab-scale A2O-activated sludge systems. Both before and after biological treatment, toxicity of treated mixture was tested.

## 2. Material and Methods

### 2.1. Leachate and Wastewater

Leachate was collected from the Municipal Solid Waste Landfill in Zabrze (Poland). There are two sections in Zabrze Landfill: I: the “old,” reclaimed section; II: the “new” one receiving municipal waste since 2007. The landfill leachate from both sections is collected separately in equalization basins and recirculated to the waste dump. Excess of leachate is pumped to the sewage collection system. The leachate flow is 80 m^3^ from each section daily. Samples were collected from the equalization basins.

Wastewater was collected from wastewater treatment plant in Zabrze-Mikulczyce (Poland). The place for wastewater collection was selected to ensure lack of earlier wastewater contamination by leachate. The daily flow of wastewater is average 5000 m^3^/d.

### 2.2. Treatment

**Figure 1:**
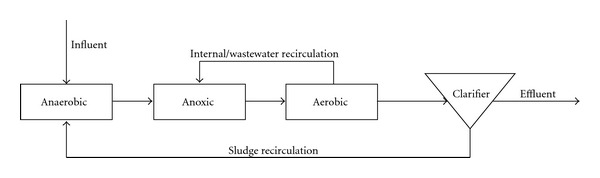
Scheme of A2O-activated sludge system.

A2O-activated sludge systems were composed of an anaerobic/anoxic/aerobic process with simultaneous nitrification-denitrification and biological phosphorus removal. Each system composed of three separate reactors with the following working volumes: anaerobic 2 L, anoxic 5 L, aerobic 7 L (Figure 1).

The experiment was carried out in three activated sludge A2O systems: I, II, and III. Influent of system I consisted of mixture of wastewater and “old” landfill leachate. Influent of system II consisted of mixture of wastewater and new landfill leachate. System III served as a control and was fed with municipal wastewater. The scheme of influents composition in different periods of experiment was presented in Table 1.

All systems were operated under the same technical parameters (Table 2) except for influent characteristic and load.

The reactors were inoculated with an activated sludge sampled from municipal wastewater treatment plant. Activated sludge in reactors I and II was acclimated to the increasing concentration of landfill leachate in the influent (1 and 10%). Some earlier study showed that median share of landfill leachate in wastewater stream should be at 5% (v/v) level [12, 13]. It was, therefore, decided that final concentration of landfill leachate in present study should not exceed 10% (v/v). After the acclimation period, systems had been operated for 8 weeks with 1% of leachates in influent. Samples for chemical analysis as well as toxicity testing were collected from average daily sample of influent/effluent. After that, leachate concentration was gradually increased to 10%.

#### 2.2.1. Chemical Analysis

Ammonium nitrogen as well as organic nitrogen was measured with Kjeltec 1026 analyzer. Chemical and biological oxygen demands (COD and BOD) were determined by standard methods [14, 15]. Chemical analysis were performed two times a week during 26 weeks research period.

### 2.3. Bioassays

Whole effluent toxicity tests were performed which means that the aggregate toxic effect of respectively influent or effluent was measured directly by a toxicity test.

Following tests were proposed for toxicity evaluation.

***Vibrio Fischeri* Luminescence Inhibition—Microtox [16]**

The test was carried out in the Microtox M500 toxicity analyzer according to the standard procedure [16], which is in accordance with ISO-DIN 38412 Part 34, 9/91. The lyophilized bacteria *Vibrio fischeri* were purchased from Azur Environmental (Carlsbad, CA, USA). As a diluents, 2% NaCl was used. As the samples of wastewater were coloured, light absorbances were measured at 490 nm and colour correction procedure was applied.

*Vibrio fischeri* luminescence inhibition test was performed three times.

***Daphnia Magna* Immobilisation Test [17]**

Tests were carried out with neonates (<24 h). Five test dilutions were prepared in a 50% dilution series for each sample with three replicates of seven animals. The test volume was 20 mL. The animals were not fed during the experiment. Each test had a duration 48 h; the temperature was 24 ± 1°C. After an exposure, the number of immobile daphnids for each dilution was recorded. *Daphnia magna* immobilization test was performed five times.

***Thamnocephalus Platyurus* Acute Toxicity Test [18]**

Tests were carried out according to the MicroBioTest Standard Operational Procedure. Readily hatched organisms were used for the test. Five test dilutions were prepared in a 50% dilution series.

Each sample was with 3 replicates of 10 animals in disposable multiwell test plates. Test volume was 1 mL per well.

After 24 h in a 25°C incubator in the dark, the number of dead crustaceans was recorded. *Thamnocephalus platyurus* acute toxicity test was performed five times.

**Freshwater Algal Growth Inhibition Test with Unicellular Green Algae [19]**

Exponentially growing *Raphidocelis subcapitata* were exposed to the test sample in batch cultures over a period of 72 hours in 24 ± 1°C. The biomass in the control cultures increased exponentially by a factor of at least 16. Five test dilutions were prepared in a 50% dilution series with an initial biomass concentration 1 × 10^4^ cells/mL. Each sample was with 3 replicates; growth inhibition test was performed five times.

## 3. Results

### 3.1. Leachate Characteristic

Chemical parameters of leachate from both sampling sites are presented in Tables 3 and 4.

### 3.2. Biological Treatment

Biological treatment of leachate has been shown to be effective in removing organic and nitrogenous matter from immature effluent characterized by high BOD/COD ratio [1, 2]. In present study biodegradability factor (BOD/COD ratio) of influents containing 1% of leachate was 0.8 and 0.6 for system I and II, respectively. BOD/COD ratio of wastewaters (system III) was 0.8. It might be, therefore, concluded that, despite 1% amendment with landfill leachate, influent of system I was similarly prone to biological degradation as wastewater (Table 5). The effluents of systems, enriched by leachate (I and II) as well as wastewater treated in system III, met the quality standards described for wastewaters introduced to surface waters or ground [20, 21]. 

Effective ammonia and organic nitrogen removal was also observed in all three systems. Removal efficiency was within the range 85–99%. 

10% amendment of leachate in wastewater stream decreased biodegradability of influent of system II (Table 5). BOD/COD ratio decreased to 0.5 for system II and remained at 0.8 level for systems I and III. Lower biodegradability of wastewater mixed with 10% of new landfill leachate resulted in high content of organic substances in effluent II (Table 6). Removal of organic content in influents of systems I and III reached, respectively, 94 and 98% BOD (78 and 83% COD. The effluent of systems enriched by new landfill leachate did not meet the quality standards described for wastewaters introduced to surface or ground waters [20, 21]. 

Combined treatment of landfill leachate and municipal wastewater was also investigated by Diamadopoulos et al. [22] in sequencing batch reactor. Parameters of the process were similar to those in the present study. The authors reported that efficiency of BOD removal was 95%, but still quality criteria were not met. 

Several authors revealed also the possibility of leachate treatment in combining aerobic-anaerobic conditions, which allowed to perform treatment with higher organic loading rates [9, 23]. Gomec et al. [24] reported combined anaerobic wastewater sludge stabilisation and treatment of landfill leachate in UASB reactor. 1% of young leachate amendment improved COD removal rate as well as biogas production. 

Organic and ammonia nitrogen was effectively removed in systems I, II, and III—removal efficiency was as high as 86, 98, and 96 percent, respectively.

### 3.3. Toxicity Testing

The results of toxicity tests were presented in Table 7 as median effect or inhibition concentrations (EC/IC_50_). 

Toxicity of new landfill leachate was significantly higher than toxicity attributed to old landfill leachate. The overall toxicity of old leachate samples allowed to classify it as toxic, while new landfill leachate toxicity was more than ten times higher, and new leachate was classified as very toxic [25]. 

The results of toxicity tests were also examined for environmental relevance by calculating toxicity units (TUs) as reported in Tables 8 and 9. The toxic unit of an effluent is the inverse of its EC_50_ (or LC_50_):

(1) $$\text{TU}=\frac{100}{\%{\text{EC}}_{50}}.$$

If the mortality in a 100% effluent concentration was between 10% and 49%, the TUs were derived as follows:

(2) TU=0.02×%  mortality.

A toxic unit of zero was allocated to mortalities between 0% and 10% in 100% effluent exposure [25, 26].

1% amendment of landfill leachate in wastewater stream slightly increased whole influent toxicity (Table 7). Significant differences versus system III are observed only towards *Daphnia magna* (system II) and *Raphidocelis subcapitata* (system I). TU values of all influents towards tested organisms were below 10; therefore, influents were classified as toxic. After biological treatment, significant reduction of toxicity was observed. Slight residual toxicity of effluent was observed only for *V. fischeri* (system I and II). All effluents stimulated growth of algae due to still high content of nutrients (nitrate and phosphates). 

Important increase of toxicity was observed in all tested bioassays while 10% of new landfill leachate was mixed with municipal wastewater (system II). 10% of old landfill leachate in wastewater stream resulted in important increase of influent toxicity towards *T. platyurus *and* V. fischeri*. Toxicity of wastewater was successfully removed during biological treatment. Significant reduction of toxicity was also observed for effluent of system II, while effluent of system I was still characterized by important residual toxicity (except for *R. subcapitata*, where growth stimulation was observed). 

Average TU values of leachate from new landfill were about 10 times higher than TU values obtained for leachate from old landfill. However, while 10% of leachate was mixed with municipal wastewater, toxicity of systems I and II influents was at similar level (Table 8). The reason of that phenomenon is that dose-response curve is usually nonlinear. Increasing toxic factor concentration might not result in similar increase of organisms' response. Similar effect was observed by Bortolotto et al. [27], where only slight, insignificant change of *Allium cepa* root length inhibition was attributed to increase of leachate concentration within the range of 40–80%. The same authors also pointed that acute toxicity of treated leachate to *Artemia salina *was very low in a range of 10–80%, even though nondiluted leachate effected in 80% mortality of crustacean. Also Bialowiec et al. [28] did not observe significant changes in *Salix amygdalina* leaf length and weight, despite exposition to landfill leachate concentration within the range of 0–12.5%. In the present study, battery of bioindicators was exposed to mixture of landfill leachate and raw wastewater. During the study, toxicity of wastewater and leachate was changing due to natural fluctuations in those samples' composition. It is commonly accepted that interaction between mixture components may result in antagonist or synergetic effects which cannot be solely predicted at the base of initial toxicity data of elements or chemical species. 

Acute toxicity of landfill leachate is often attributed to high ammonium nitrogen concentration [6, 29, 30]. In the present study, however, residual toxic effect was observed for system I effluent towards 3 (out of 4) tested organisms even though ammonium nitrogen was successfully removed during biological treatment. In that case residual toxicity of system I treated wastewater was caused by recalcitrant organic compounds, which were not removed during the treatment. Biological cotreatment of leachate from old landfill and municipal wastewater could not be, therefore, suggested as safe method for landfill leachate toxicity reduction. 

In case of leachate from new landfill—at the base of present and some previous studies [31, 32]—dose-response curve was derived for biologically cotreated leachate:

(3) $$y=0.7752\cdot e^{0.0681x}.$$

Investigation was performed for 1, 5, 10, and 15% of new landfill leachate cotreated with municipal wastewater. After biological treatment, toxic response of effluents significantly differed from the control for wastewater containing 5, 10, and 15%. of leachate. The maximum concentration of leachate, which, after biological cotreatment, would not be hazardous for more than 5% of species, could be roughly assessed with use of safety factors. For the toxicity measurement, except from *Raphidocelis subcapitata* growth inhibition test, only acute toxicity tests were performed. Moreover, small group of 4 organisms served as biotest battery. Therefore, counting the hazardous concentration of landfill leachate, safety factor of 100 should be used. The highest leachate concentration, which after biological treatment would not be harmful for 95% of aquatic species, is 0.05%. 

## 4. Conclusions

Landfill leachate significantly disrupts biological treatment of wastewater. After biological treatment, wastewater enriched with 10% leachate did not meet the water quality standards and still was harmful to aquatic organisms. 

The calculated concentration of new landfill leachate, which after biological treatment would not be harmful for aquatic organisms, was 0. 05%. 

## Figures and Tables

**Table 1:** Composition of influent of systems I, II, and III.

System	Type of landfill	Volume of leachate [%] (v/v)	Volume of wastewater [%] (v/v)
I	Old	1	99
		10	90
II	New	1	99
		10	90
III	—	0	100

**Table 2:** Operational parameters of activated sludge systems I, II, and III.

Parameter	Unit	System	Range	Average ± SD	Median
Sludge loading rate	g COD/ g MLSS*·*d	I: 1%	0.08–0.13	0.10 ± 0.01	0.10
		I: 10%	0.11–0.15	0.11 ± 0.02	0.11
		II: 1%	0.07–0.236	0.08 ± 0.02	0.10
		II: 10%	0.08–0.23	0.15 ± 0.03	0.17
		III	0.06–0.175	0.09 ± 0.04	0.06
MLSS	g/L	I			
		II	1.6–2.5	2.0 ± 0.2	2.0
		III			
Q	L/d	I			
		II	8.5–10.0	9.5 ± 0.5	9.7
		III			
HRT	d	I		1.5 ± 0.1	1.4
		II	1.4–1.6	1.6 ± 0.4	1.5
		III		1.5 ± 0.1	1.5

SD: standard deviation; number of measurements *n* = 16; time-dependent variation caused by unstable composition of influent.

COD: chemical oxygen demand.

MLSS: activated sludge concentration.

Q: wastewater flow.

HRT: hydraulic retention time.

**Table 3:** Characteristic of “old” landfill leachate (nondiluted).

Parameter	Unit	Range	Average ± SD*	Median
TOC	mg/L	309–352	327 ± 21	324
COD	mg/L	381–435	403 ± 19	400
BOD	mg/L	120–150	134 ± 11	130
N NH4	mg/L	26–60	43 ± 10	52

*SD: standard deviation; number of measurements  *n* = 16; time-dependent variation of parameters value was caused by unstable composition of leachate.

**Table 4:** Characteristic of “new” landfill leachate (nondiluted).

Parameter	Unit	Range	Average ± SD*	Median
TOC	mg/L	1460–2300	1950 ± 350	2010
COD	mg/L	1873–3600	2560 ± 615	2330
BOD	mg/L	150–273	210 ± 55	210
N NH4	mg/L	971–1250	1100 ± 92	1200

*SD: standard deviation; number of measurements *n* = 16; time-dependent variation of parameters value was caused by unstable composition of leachate.

**Table 5:** Chemical characteristics of raw and treated wastewater (1% of leachate in influent I and II).

Parameter		Unit	Range	Average ± SD	Percentile 80/100	Range	Average ± SD	Percentile 80/100	Mean removal [%]
			Influent			Effluent			
	I		265–346	314 ± 41	344	57–124	86 ± 24	106	72
COD	II	mg/dm^3^	213–486	316 ± 84	346	15–142	112 ± 38	138	64
	III		153–356	222 ± 71	263	46–90	65 ± 15	80	83
	I		230–270	248 ± 15	246	5–10	8 ± 3	10	97
BOD	II	mg/dm^3^	180–200	190 ± 10	190	10–20	13 ± 6	10	93
	III		160–200	180 ± 20	240	10–20	13 ± 6	16	93
	I		106–151	139 ± 21	152	19–34	21 ± 6	26	85
N_og_	II	mg/dm^3^	87–226	161 ± 68	217	1–9	3 ± 2	4	98
	III		73–181	116 ± 40	150	1–4	2 ± 1	1.0	99
	I		98–124	97 ± 10	118	1–3	3 ± 2	5	97
N-NH4	II	mg/dm^3^	85–177	128 ± 29	154	1–9	2 ± 2	4	98
	III		53–131	78 ± 20	87	0–3	1 ± 1	2	99

**Table 6:** Chemical characteristics of raw and treated wastewater (10% of leachate in influent I and II).

Parameter		Unit	Range	Average ± SD	Percentile 80/100	Range	Average ± SD	Percentile 80/100	Mean removal [%]
			Influent			Effluent			
	I		300–410	348 ± 47	390	43–114	74 ± 24	90	78
COD	II	mg/dm^3^	281–650	460 ± 95	520	62–233	150 ± 45	182	67
	III		257–362	316 ± 49	361	34–83	55 ± 22	70	83
	I		240–280	262 ± 18	280	10–20	17 ± 6	20	94
BOD	II	mg/dm^3^	240 + 270	250 ± 17	258	10–20	10 ± 0	10	96
	III		240–280	264 ± 17	272	0–10	6.0 ± 4	8	98
	I		87–151	109 ± 41	133	8–23	16 ± 6	21	86
N_og_	II	mg/dm^3^	135–380	277 ± 66	315	4–9	6 ± 2	6	98
	III		73–296	150 ± 71	200	0–13	6 ± 4	10	96
	I		26–70	53 ± 16	61	2–10	5 ± 3	8	90
N-NH4	II	mg/dm^3^	128–255	200 ± 33	222	3–16	6 ± 3	7	97
	III		80–160	120 ± 26	136	2–10	5 ± 4	8	96

**Table 7:** Results of landfill leachate toxicity tests (average values from three experiments).

Organism	Old landfill leachate		New landfill leachate	
	EC/IC_50_ [%]	TU ± SD	EC/IC_50_ [%]	TU ± SD
*Thamnocephalus platyurus*	98 ± 7.0	1.0 ± 0.1	1.4 ± 0.2	71.4 ± 9.0
*Daphnia magna*	max⁡. effect 35% ± 3.0	0.7 ± 0.06	2.6 ± 0.6	38.5 ± 9.2
*Vibrio fischeri*	28 ± 5.6	3.6 ± 0.7	2.8 ± 0.3	36.0 ± 4.0
*Raphidocelis subcapitata*	67 ± 13.4	1.5 ± 0.3	3.0 ± 0.3	34.0 ± 3.7

**Table 8:** Toxicity of raw and treated wastewater (1% of leachate in influent I and II; the 95% confidence limit in parenthesis).

Organism	Number of tests	System	Influent		Effluent	
			Average EC_50_ [%] ± SD	TU	Average EC_50_ [%] ± SD	TU
*Daphnia magna*	5	I	33.0 (23.3–42.7)	3.2 ± 0.8	h.e. < 10%	0
	5	II	25.5 (22.6–28.7)*	4.0 ± 0.5	h.e. < 10%	0
	5	III	36.3 (28.4–44.2)	2.8 ± 0.6	h.e. < 10%	0
*Thamnocephalus platyurus*	5	I	18.0 (10.1–25.9)	7.2 ± 4.4	h.e. < 10%	0
	5	II	16.3 (10.1–22.5)	7.9 ± 4.0	h.e. < 10%	0
	5	III	18.8 (16.4–21.2)	7.5 ± 4.2	0	0
*Raphidocelis subcapitata*	5	I	67.0 (53.9–80.1)*	1.5 ± 0.3	Growth stimulation	0
	5	II	44.0 (28.7–59.2)	2.6 ± 1.0		0
	5	III	54.0 (42.4–65.6)	1.9 ± 0.6		0
*Vibrio fischeri*	5	I	23.5 (19.2–27.9)	4.3 ± 0.6	h.e. 20%*	0.4 ± 0.2
	5	II	24.3 (19.9–28.6)*	4.2 ± 0.6	91.5 (80.1–102.9)*	0.8 ± 0.5
	5	III	21.0 (14.8–27.2)	5.0 ± 1.2	0	0

h.e.: highest observed effect for nondiluted sample.

*Indicate significant differences versus control III (Students *t*-test, *P* < 0.05).

**Table 9:** Toxicity of raw and treated wastewater (10% of leachate in influent I and II; the 95% confidence limit in parenthesis).

Organism	Number of tests	System	Influent		Effluent	
			Average EC_50_ [%]	TU ± SD	Average EC_50_ [%]	TU ± SD
*Daphnia magna*	5	I	32 (15.2–48.8)	4.3 ± 3.1	h.e. 28%	0.6 ± 0.2
	5	II	23.6 (21.7–25.5)*	4.3 ± 0.4	0 (h.e. < 10%)	0
	5	III	43.7 (38.1–49.3)	2.3 ± 0.3	0	0
*Thamnocephalus platyurus*	5	I	10.6 (7.8–13.4)*	10.7 ± 4.1	84 (76.9–91.1)*	1.1 ± 0.2
	5	II	10.7 (9.4–12.0)*	9.4 ± 1.1	0 (h.e. < 10%)	0
	5	III	19.8 (17.3–22.3)	5.1 ± 0.7	0	0
*Raphidocelis subcapitata*	5	I	57.0 (47.5–66.5)	1.8 ± 0.3	Growth stimulation	0
	5	II	32.3 (31.6–33.0)*	3.2 ± 0.8		0
	5	III	55.0 (52.1–57.9)	1.8 ± 0.1		0
*Vibrio fischeri*	3	I	17.1 (11.3–22.9)*	6.2 ± 1.4	55.9 (30.7–80.3)*	1.8 ± 0.5
	3	II	15.3 (12.2–18.4)*	6.6 ± 1.1	87.3 (82.1–92.5)*	1.1 ± 0.1
	3	III	30.3 (27.2–33.4)	3.3 ± 0.3	0	0

h.e.: highest observed effect for nondiluted sample.

*Indicates significant differences versus control-III (Students *t*-test, *P* < 0.05).
